# Let's reduce the blood volume collected for laboratorial exams?

**DOI:** 10.1590/0103-058220143221613

**Published:** 2014-06

**Authors:** Magda Carneiro-Sampaio, Natasha Slhessarenko

**Affiliations:** 1 Faculdade de Medicina da USP, São Paulo, SP, Brasil; 2 Faculdade de Medicina da Universidade Federal de Mato Grosso (UFMT)

The spoliation due to blood collection for laboratory testing is currently the leading cause of transfusion in neonates and infants with prolonged hospitalization in Brazilian hospitals^(^ 1 ^)^. This is a worrying situation that needs to be reversed in our pediatric health services, particularly in neonatal care.

Paradoxically, the volume of blood required to perform most analyzes (hematology, biochemical, serology, hormonal, and molecular techniques) is presently very small and rarely exceeds 200µL (0.2mL), already considering a repetition of the exam. To perform biochemical, hormonal, and serology exams, the required volume ranges from 2 to 100µL for each test. However, to conduct a complete blood count (CBC), which is performed nowadays in a flow cytometer, only 100µL of whole blood are required. Nevertheless, in a standard procedure, 4,5mL are collected, that is, a volume that is sufficient to perform not one, but 45 whole CBCs! This is only an example of an ordinary laboratory test which shows how the analytical phase itself has advanced regarding the so-called pre-analytical phase (specimen collection and sample preparation), whose practice needs to be urgently reviewed in our laboratories, especially those that provide neonatal and pediatric services in general. 

Let us consider the hypothetical case of a hospitalized child weighing 3 kg and who needs to be submitted every 2 or 3 days to a hematologic evaluation. A volume of 4.5mL represents approximately 1.8% of its volume (estimated at 250mL) and would rise to 3% in the case of a preterm weighing 1.5kg (blood volume of about 150mL)^(^ 2 ^)^. Applying these prepositions to an adult of 70kg, this volume would correspond to 90 and 150mL...only for the CBC! 

The increasing automation of modern laboratories allows that increasingly accurate analyzes be performed with reduced volumes of blood, serum, or other fluids^(^ 3 ^)^. It is also noteworthy that financial constraints to acquire modern laboratory equipment are progressively decreasing, as its use on commodate-loan is the rule nowadays. 

On the other hand, regarding the pre-analytical phase, the use of micro-collectors is still a practice restricted to elite private services and University Hospitals located in advanced medical centers. The micro-collection requires special tubes that also have a higher cost than the traditional ones. However, the differences in price are not so great and could be easily minimized by the acquisition of large batches of tubes, with wide adherence to the techniques of micro-collection. With the implementation of the program "Child-friendly Diagnosis (Diagnóstico Amigo da Criança)," the Child Institute at Hospital das Clínicas within the Medical School of Universidade de São Paulo, for instance, has had an additional cost of R$ 8,000 monthly for the acquisition of microtubes, aiming to serve 214 beds of severe and complex cases^(^ 4 ^)^. These costs are largely justified by the reduction of risks of blood transfusions and are covered by the financial savings from the reduction of hemotherapeutic procedures. Not to mention the reduction in life risk for our little patients. 

Long before implanting measures aiming to reduce the blood volume for Laboratory analyzes, it is necessary to encourage the rationalization in the request of complementary exams, both in the area of clinical pathology and in relation to imaging and functional specialized exams. Our Medicine today increasingly prioritizes what is classically called supplementary exam to the detriment of the clinical data collection, by means of anamnesis and careful physical examination. We urgently need to promote a recovery of clinics and to encourage good practices in the request of complementary exams, with awareness regarding the contribution that each exam will bring for the diagnosis or the conduction of the case. At the same time, it is necessary to discourage the request of exams into "batteries" and "packs" proposed for given clinical situations. It is urgent that clinical rationalization be prioritized and that complementary exams start to play their real role again. 

With this text, we want to claim to all pediatricians, especially neonatologists and intensivists, to get involved with the measures proposed to limit the amount of blood collected to what is really necessary to perform the analysis. Furthermore, we suggest a higher integration between the teams at pediatric units and the laboratory staff, so that they are always aligned regarding the difficulties to obtain samples and the possibility to make the most of all blood volume to conduct the tests. The laboratory professionals, in turn, should be always sensitive towards the needs and complaints of child health units. We call everyone to engage in this fight so that pediatric blood collection becomes a reality all over the country. After all, the mortality rates in Brazil are still high, especially in the perinatal and neonatal components, and all measures that may promote this reduction, even if in a small scale, should be adopted.
