# Supplementary material for: Let's reduce the blood volume collected for laboratorial exams?
Source: Rev Paul Pediatr. 2014 Jun;32(2):291–2. doi: 10.1590/0103-058220143221613 (PMC4183028; doi:10.1590/0103-058220143221613)
Supplement: Translation

# Translation: Vamos reduzir o volume de sangue colhido para exames laboratoriais?

**Authors:** Magda Carneiro-Sampaio, Natasha Slhessarenko

**Affiliations:** 1 Faculdade de Medicina da USP, São Paulo, SP, Brasil; 2 Faculdade de Medicina da Universidade Federal de Mato Grosso (UFMT)

A espoliação devido à coleta de sangue para análises laboratoriais representa hoje a principal causa de transfusão em recém-nascidos e lactentes com internações prolongadas nos hospitais brasileiros^(^ ^1^ ^)^. Essa é uma situação preocupante e que precisa ser revertida em nossos serviços de saúde pediátricos, em particular, nos neonatais.

Paradoxalmente, o volume de sangue necessário para realizar a maior parte das análises (hematológicas, bioquímicas, sorológicas, hormonais e por técnicas moleculares) é hoje muito pequeno e raramente ultrapassa 200µL (0,2mL), já se considerando uma repetição do exame. Para se efetuarem exames de bioquímica, hormônios e sorologia, o volume necessário varia de 2 a 100µL para cada teste. Já para se realizar um hemograma completo, feito hoje em citômetro de fluxo, são requeridos apenas 100µL de sangue total. No entanto, em um procedimento padrão, coletam-se 4,5mL, ou seja, um volume suficiente para se realizar não um, mas 45 hemogramas! Esse é apenas um exemplo de exame laboratorial corriqueiro e que demonstra como a fase analítica propriamente dita avançou quanto à chamada fase pré-analítica (coleta do material e preparação da amostra), cuja prática precisa ser urgentemente revista em nossos laboratórios, especialmente naqueles que atendem os serviços neonatais e pediátricos em geral. 

Analisemos o caso hipotético de uma criança de 3kg internada e que precisa ser submetida a cada dois ou três dias a uma avaliação hematológica. Um volume de 4,5mL representa aproximadamente 1,8% de sua volemia (estimada em 250mL) e subiria para 3% caso se tratasse de um prematuro de 1,5kg (volemia de cerca de 150mL)^(^ ^2^ ^)^. Transpondo essas proporções para um adulto de 70kg, esse volume corresponderia a 90 e 150mL... só para o hemograma! 

A crescente automação dos laboratórios modernos permite que mais e mais análises precisas sejam realizadas com volumes reduzidos de sangue, soro ou outros fluidos^(^ ^3^ ^)^. Cabe também salientar que as limitações financeiras para adquirir modernos equipamentos laboratoriais são cada vez menores, pois o seu uso em comodato representa hoje a regra. 

Por outro lado, quanto à fase pré-analítica, a utilização de microcoletores ainda é uma prática restrita a serviços privados de elite e a hospitais universitários localizados em centros médicos avançados. A microcoleta requer tubos especiais e que ainda têm um custo mais elevado que os tradicionais. Entretanto, as diferenças de preço não são tão grandes e poderiam ser facilmente minimizadas pela aquisição de grandes lotes de tubos, com aderência ampla às técnicas de microcoleta. Com a implantação do programa "Diagnóstico Amigo da Criança", o Instituto da Criança do Hospital das Clínicas da Faculdade de Medicina da Universidade de São Paulo, por exemplo, tem tido um ônus adicional de R$ 8.000,00 mensais para a aquisição de microtubos, visando atender seus 214 leitos de casos graves e complexos^(^ ^4^ ^)^. Esses custos são amplamente justificados pela redução dos riscos das transfusões de sangue e cobertos pela economia financeira com a redução de procedimentos hemoterapêuticos. Isso para não falar da redução nos riscos para a vida dos nossos pequenos pacientes. 

Muito antes de implantar medidas visando à redução do volume de sangue para análises de laboratório, é preciso estimular a racionalização na solicitação dos exames complementares, quer na área da patologia clínica, quer em relação aos exames de imagem e funcionais especializados. A nossa Medicina de hoje assiste cada vez mais à priorização do que classicamente é chamado de exame complementar em detrimento da coleta de dados clínicos, por meio da anamnese e do exame físico cuidadosos. Precisamos promover urgentemente um resgate da clínica e estimular as boas práticas na solicitação de exames complementares, com a conscientização sobre a contribuição que cada exame solicitado trará para o diagnóstico ou para a condução do caso. Ao mesmo tempo, é preciso desestimular a solicitação em "baterias" e "pacotes" propostos para determinadas situações clínicas. É urgente que o raciocínio clínico seja priorizado e que os exames complementares voltem a exercer o seu real papel.

Com este texto, queremos apelar a todos os pediatras, em especial aos neonatologistas e intensivistas, para que se envolvam com as medidas propostas para reduzir a quantidade de sangue coletada àquela realmente necessária para realizar a análise. Além disso, sugerimos que haja uma maior integração entre as equipes das unidades pediátricas e as do laboratório, de modo que estejam sempre alinhadas sobre as dificuldades de obtenção das amostras e a possibilidade do aproveitamento integral de todo o volume de sangue para realizar os testes. Já os profissionais do laboratório devem mostrar-se sempre sensíveis às necessidades e queixas das unidades infantis. Pedimos a todos que se engajem nessa luta para que a coleta de sangue pediátrica se torne uma realidade em todos os cantos deste país. Afinal, as taxas de mortalidade infantil no Brasil ainda são elevadas, sobretudo nos componentes perinatal e neonatal, e todas as medidas que possam promover sua redução, ainda que em pequena escala, devem ser adotadas.
